# CXCR1 and CXCR2 Antagonism with G31P Attenuates Chemotherapy-Induced Lung Inflammation and Augments the Gefitinib Therapeutic Response in Lung Cancer

**DOI:** 10.32604/or.2025.069408

**Published:** 2025-11-27

**Authors:** Muhammad Noman Khan, Kang Tian, John R. Gordon, Fang Li, Song-Ze Ding

**Affiliations:** 1 Henan University People’s Hospital, Henan Provincial People’s Hospital, Zhengzhou, 450003, China; 2 College of Basic Medical Sciences, Dalian Medical University, Dalian, 116011, China; 3 Unani Medicine Research Laboratory, Faculty of Eastern Medicine, Hamdard University, Karachi, 74600, Pakistan; 4 Division of Respirology, Critical Care and Sleep Medicine, Royal University Hospital, University of Saskatchewan, Saskatoon, SK S7N 5E5, Canada

**Keywords:** CXC chemokine receptor 1/2, G31P, gefitinib, pulmonary inflammation, lung cancer, pneumonitis

## Abstract

**Objectives:**

Chemotherapy-induced lung inflammation limits the efficacy of anticancer therapies such as gefitinib in non-small cell lung cancer (NSCLC). Glutamic acid-leucine-arginine positive (ELR+) CXC chemokines and their receptors, CXC chemokine receptor 1 and 2 (CXCR1 and CXCR2), mediate both inflammatory responses and tumor progression. This study evaluated the effects of CXCR1/2 antagonism by G31P, a CXC motif chemokine ligand 8 (CXCL8)-mutated peptide, alone or in combination with gefitinib, on lung cancer growth and chemotherapy-induced pulmonary inflammation.

**Methods:**

Human NSCLC cell lines (A549 and H460) were treated with gefitinib and/or G31P. Cell proliferation, apoptosis, and signaling pathways, including protein kinase B (AKT) and extracellular signal-regulated kinase (ERK) phosphorylation, were evaluated by cell counting kit-8 (CCK-8) assay, flow cytometry, and Western blotting. An orthotopic lung tumor xenograft model was established in BALB/c nude mice to evaluate tumor growth, metastasis, cytokine expression, and lung histopathology. A bleomycin-induced lung injury model was also used to assess the anti-inflammatory effects of G31P, with or without gefitinib, by quantitative reverse transcription polymerase chain reaction (qRT-PCR) and flow cytometry of inflammatory markers.

**Results:**

G31P and Gefitinib, either alone or combined, inhibited proliferation and migration of A549 and H460 cells *in vitro*. Combination treatment effectively reduced AKT and ERK phosphorylation in both cell lines. *In vivo*, G31P with gefitinib significantly suppressed tumor growth, metastasis, and increased apoptosis. G31P decreased CXCL1 and CXCL2, and tumor necrosis factor-alpha (TNF-α) mRNA levels, lung hydroxyproline content, and myeloperoxidase (MPO) activity in the lungs of mice. In the bleomycin-induced lung injury model, G31P similarly reduced inflammatory responses.

**Conclusion:**

CXCR1/2 antagonism by G31P attenuates chemotherapy-induced pulmonary inflammation and enhances the anti-tumor efficacy of gefitinib in NSCLC. These findings support the therapeutic potential of G31P as an adjuvant to epidermal growth factor receptor tyrosine kinase inhibitors (EGFR-TKIs) to improve clinical outcomes by limiting inflammation.

## 1 Introduction

Chemotherapy remains the cornerstone of cancer treatment for many malignancies. However, its clinical use is often limited by serious side effects, including toxicity to healthy cells and tissues, and ultimately results in chemoresistance [1]. A major goal of oncology research is to uncover the mechanisms underlying these adverse effects and to identify strategies to mitigate them [2]. One such approach involves combining drugs with synergistic or additive effects, which may improve therapeutic efficacy while reducing the required dosage and associated toxicity of each drug [3].

Gefitinib (also known as Iressa or ZD1839) is an epidermal growth factor (EGFR) tyrosine kinase inhibitor (TKI) widely used to treat advanced non-small cell lung cancer (NSCLC) [4]. Besides its clinical importance, gefitinib treatment is also associated with adverse effects, including diarrhea, skin rashes, and particularly interstitial lung disease (ILD) [5,6]. Preclinical studies have reported that gefitinib exacerbates pulmonary inflammation and increases lung toxicity by promoting immune cell infiltration and upregulation of inflammatory cytokines in irradiated mice [7]. Clinically, high doses of gefitinib are linked to an increased risk of ILD, especially in Asian populations [8]. Risk factors for gefitinib-induced ILD include smoking history and pre-existing ILD or pulmonary fibrosis [9]. However, the underlying molecular mechanisms driving this toxicity remain poorly understood.

Chemotherapy-induced lung injury, including pneumonitis and fibrosis, is driven by pro-inflammatory mediators produced by epithelial, endothelial, and immune cells. Among them, Glutamic acid-leucine-arginine positive (ELR+) CXC chemokines such as CXC motif chemokine ligand (CXCL) 1-3, 5, 6, and 8 are important in neutrophil and macrophage infiltration into injured tissues [10]. CXCL6 and 8 signal through CXC chemokine receptor 1/2 (CXCR1/2), while CXCL1-3 and CXCL5 bind primarily to CXCR2 [11]. These cytokines mediate inflammation and are also involved in the development of many tumor types. Elevated CXCL8 levels are associated with poor prognosis in various cancers, including NSCLC [12–14]. CXCL8 promotes tumor cell proliferation, angiogenesis, and chemoresistance [15,16], while neutrophil infiltration mediated by CXCR1/2 correlates with poor clinical outcomes [13].

Targeting the CXCL8-CXCR1/2 axis represents a promising anti-inflammatory and anti-tumor strategy. In NSCLC, CXCL1 and 8 are particularly important ELR+ CXC chemokines involved in both tumor progression and drug-induced inflammation. Their signaling via CXCR1/2 contributes to neutrophil infiltration and paracrine signaling which sustains the inflammatory tumor microenvironment [17]. Pharmacological approaches to block CXCR1/2 signaling include small molecule inhibitors, monoclonal antibodies, and modified chemokine therapies with high affinity for receptor blockade [18]. G31P is a synthetic variant of CXCL8 [CXCL8(3-72)K11R/G31P] containing amino acid substitutions (K11R and G31P) that confer selective antagonism of both CXCR1/2 [19]. G31P has shown anti-tumor activity in several epithelial cancers, including prostate, liver, and lung, and has also demonstrated efficacy in mitigating inflammation in various inflammatory diseases [20]. However, its role in managing drug-induced pulmonary inflammation or improving the therapeutic efficacy of EGFR-TKIs such as gefitinib has not been investigated.

In this study, we evaluated the therapeutic potential of G31P in NSCLC, focusing on both tumor suppression and mitigation of gefitinib-induced lung inflammation. Using *in vitro* and *in vivo* mouse models, the role of G31P was investigated for its ability to enhance anti-tumor responses and reduce gefitinib-induced lung inflammation. This study enhances our understanding of the interplay between inflammation and cancer treatment and may help to identify new approaches to improve outcomes in NSCLC patients receiving EGFR-targeted therapy.

## 2 Materials and Methods

### 2.1 Cell Culture

Human non-small cell lung cancer (NSCLC) cell lines, H460, A549, H1299, and H1650 were obtained from American Type Culture Collection (ATCC; Manassas, VA, USA). All cell lines were authenticated using short tandem repeat profiling within last six months by the supplier and to ensure experimental reliability, Mycoplasma contamination was regularly tested by PCR-based assay and confirmed negative prior to use. Cells were cultured in RPMI-1640 medium (Cat# SH30809.01, HyClone^TM^, Logan, UT, USA) supplemented with 10% fetal bovine serum (FBS; Cat# 10099141, Gibco, Thermo Fisher Scientific, Waltham, MA, USA) and 1% penicillin-streptomycin (Cat# 15140122, Gibco). All cell cultures were maintained at 37°C in a humidified incubator with 5% CO_2_. The recombinant protein G31P, a CXCR1/2 inhibitor, was kindly provided by Prof. Dr. Jya-Wei Cheng (National Tsing Hua University, Hsinchu, Taiwan).

### 2.2 Cell Proliferation Assay

Cell viability was quantified using the Cell Counting Kit-8 (CCK-8) assay kit (Cat# CK04; Dojindo Laboratories, Kumamoto, Japan). NSCLC cells (A549, H460, H1299, and H1650) were plated in 96-well plates at 5000 cells/well and incubated for 12 h in RPMI-1640 medium containing 10% FBS. Gefitinib (cat# S1025, Selleck Chemicals, Houston, TX, USA) with dosage of 0.1, 1, 2 or 5 μM was added with or without G31P (100 ng/mL). After 48 h of incubation at 37°C with 5% CO_2_, media were supplemented with 10 μL CCK-8 solution in 110 μL RPMI and incubated for 4 h with a final dilution of 1:11 (10 μL CCK-8 in 110 μL medium). Absorbance was read at 450 nm using a Bio-Rad iMark Microplate Absorbance Reader (Model # 1681135, Bio-Rad Laboratories, Hercules, CA, USA). All conditions were tested in triplicate.

### 2.3 Ki-67 Immunostaining

A549 and H460 cells were seeded at a density of 1 × 10^5^ cells/well on coverslips in 6-well plates and subsequently with gefitinib (5 μM), both with or without G31P (100 ng/mL) for 24 h. Following treatment, cells were fixed with 4% paraformaldehyde for 20 min, permeabilized with 0.2% Triton X-100 in PBS (pH = 7.4, 1×) for 10 min. After blocking with 2% BSA for 30 min at 37°C. Cells were incubated with primary antibody targeting Ki-67 (Cat# 550609, mouse monoclonal, dilution 1:100, BD Biosciences, San Jose, CA, USA) for 30 min, followed by PE-conjugated secondary antibody (Cat# P9670, dilution 1:100, Sigma-Aldrich, St. Louis, MO, USA) for 30 min. After thorough rinsing with PBS, the coverslips were mounted with DAPI-containing Mowiol media. Imaging was performed with an Olympus BX-51 fluorescence microscope (Model No. BX51 TR32000; Olympus Corporation, Tokyo, Japan) and signal quantification was acquired using ImageJ v1.46r software (NIH, Bethesda, MD, USA).

### 2.4 Wound Healing Assay

A549 and H460 cells were seeded at 2 × 10^5^ cells/well in 12-well plates to approximately 80% confluence. A scratch was made with a 200 μL pipette tip, washed with phosphate buffer saline (PBS; pH = 7.4, 1×) to remove debris. Cells were treated with gefitinib (5 μM) with or without G31P (100 ng/mL) in complete medium and incubated for 24 h at 37°C and 5% CO_2_. Migration into the wound area was monitored at 0 and 24 h microscopically using Leica DMi8 inverted microscope (Model DMi8, Leica Microsystems, Wetzlar, Germany), and gap closure was quantified by measuring the distance between wound edges.

### 2.5 Transwell Migration Assay

To evaluate migratory potential, A549 and H460 cells pre-treated with gefitinib and/or with G31P were seeded (1.5 × 10^4^ cells/well) in serum-free medium in the upper chambers of transwell inserts (8 μm pores; Corning, NY, USA). The lower chambers were filled with 10% FBS (pH = 7.4) medium and after 24 h, non-migrated cells were removed, while transmigrated cells on the underside were fixed with 3.5% formalin for 10 min and stained with 0.1% crystal violet for 30 min. Migrated cells were visualized and counted with 100× magnification using Leica DMi8 inverted microscope (Model DMi8, Leica Microsystems, Wetzlar, Germany).

### 2.6 Western Blot Analysis

Proteins were extracted from A549 and H460 cells using RIPA lysis buffer (Cat# P0013B, Beyotime Biotechnology, Shanghai, China) supplemented with protease and phosphate inhibitor cocktail (Cat# P1045, Beyotime). After incubation on ice for 30 min, lysate was centrifuge at 12,000× *g* for 15 min at 4°C, and the supernantants were collected. Protien concentration was determined using BCA Protein Assay Kit (Cat# P0010, Beyotime). Equal amount of protien were loaded and separated on SDS-PAGE, then transferred to PVDF membranes. After blocking, membranes were probed overnight at 4°C with primary antibodies against AKT (Cat# 9272, 1:1000, Cell signaling Technology (CST), Danvers, MA, USA), pAKT (Ser473) (Cat# 9271, 1:1000, CST), ERK1/2 (Cat# 4695, 1:1000, CST), pERK1/2 (Cat# 4370, 1:1000, CST), and GAPDH (Cat# 10494-1-AP, 1:1000, Proteintech Group Inc., Rosemont, IL, USA). Secondary HRP-conjugated anti-rabbit or anti-mouse antibodies (Cat# SA100001-1 or SA100001-2, 1:4000, Proteintech) were incubated with membrane overnight at 4°C, and raised with PBS. Detection was performed using ECL HRP Substrate (Cat# K-12045-D10, Advansta, San Jose, CA, USA), and protein bands were visualized on a ChemiDoc MP Imaging System (Model # 1708280, Bio-Rad Laboratories, Hercules, CA, USA). Band densitometry was collected using ImageJ software version 1.46r (NIH, Bethesda, MD, USA) and analysed subsequently.

### 2.7 Orthotopic Lung Tumor Xenograft Model

Female BALB/c nude mice (4–6 weeks old, weight 18–20 g) were obtained from Specific Pathogen-Free (SPF) Facility Centre of Dalian Medical University (Dalian, China) and were housed under SPF conditions with controlled temperature (25°C ± 3°C) and humidity (55% ± 10%), and a 12 h light/dark cycle, with *ad libitum* access to food and water. To establish orthotopic lung tumors, donor BALB/c mice were subcutaneously injected with 5 × 10^6^ H460-GFP cells in 0.2 mL PBS (pH = 7.4). When tumors reached 1–1.5 cm in diameter, they were aseptically excised and cut into 1–2 mm^3^ pieces. These tumor fragments were surgically implanted into the right lung of recipient mice (n = 24) under isoflurane anesthesia following guidelines of Dalian Medical University Animal Welfare and Ethics Review Committee (Approval No. 202310261). One week post-implantation, mice were randomized into 4 groups (n = 6 per group). Treatment comprised oral administration of gefitinib (300 mg/kg, five times per week) with or without intraperitoneal (i.p.) injections of G31P (0.5 mg/kg) on alternate days, for a duration of 28 days. Investigators were blinded to group allocation during outcome assessments. At the study endpoint, mice were euthanized by CO_2_ inhalation followed by cervical dislocation. Tumor burden and GFP-labeled metastatic spread were evaluated. Tumors were resected for subsequent tumor weight, tumor volume, histological and molecular analysis. Tumor volume in mm^3^ was calculated as: tumor volume = (length × width^2^)/2.

### 2.8 TUNEL Assay

Apoptotic cells in tumor tissue sections were assessed using TUNEL staining kit (RFP-TUNEL Apoptosis detection Kit, Cat# KGA710, KeyGEN Biotechnology, Nanjing, China). Tissue sections were deparaffinized, digested with proteinase K (20 μg/mL, 15 min, 37°C), and rinsed. Sections were incubated with RFP-labeled dUTP solution for 1 h at 37°C. Fluorescent signals were captured using an Olympus BX-51 microscope (Model No. BX51 TR32000; Olympus Corporation, Tokyo, Japan). Apoptotic cells were quantified based on red fluorescence. TUNEL-positive apoptotic cells were counted in five randomly selected high fields per section and apoptotic index was calculated as (TUNEL-positive cells/GFP-positive cells) × 100. ImageJ v1.46r (NIH) software was used for cell counting and analysis.

### 2.9 PCNA Immunohistochemistry

Paraffin-embedded tumor sections (5 μm) were deparaffinized, rehydrated, and subjected to antigen retrieval in citrate buffer (10 mM, pH = 6.0) for 20 min. Endogenous peroxidase activity was blocked with 3% hydrogen peroxidase for 10 min at room temperature, followed by blocking with 5% bovine serum albumin (BSA) for 30 min. Sections were incubated overnight at 4°C with primary antibody against proliferating cell nuclear antigen (PCNA; Cat# 10205-2-AP, 1: 200, Proteintech, Rosemont, IL, USA). After washing with PBS (pH = 7.4), slides were incubated with HRP-conjugated goat anti-rabbit IgG secondary antibody (Cat# SA00001-2, 1:4000, Proteintech, Rosemont, IL, USA), developed with DAB substrate kit (ZSBG-Bio, Beijing, China), counterstained with hematoxylin, and imaged using an BX-51 fluorescence microscope (Model No. BX51 TR32000; Olympus Corporation, Tokyo, Japan).

### 2.10 Bleomycin-Induced Pulmonary Fibrosis

Male C57BL/6 mice (4–6 weeks old, weight 18–20 g) were obtained from SPF center of Dalian Medical University (Dalian, China), and were anesthetized with 3% isoflurane and intraperitoneal etomidate (Cat# HY-13683, MedChem Express, Monmouth Junction, NJ, USA; 40–80 mg/kg). A tracheal incision allowed bleomycin sulfate (Cat# 42291, Nippon Kayaku Co., Ltd., Tokyo, Japan; 4 U/kg) delivery via a 27-G needle into the trachea. Thoracic compression-assisted deep lung distribution. Skin incisions were closed post-procedure, and mice were allowed to recover. Gefitinib was administered orally at 300 mg/kg body weight, five times per week and G31P was given by i.p. injections on alternate days for 14 days. At the end of treatment, mice were euthanized by CO_2_ inhalation followed by cervical dislocation. Lungs from mice were harvested for further histology and molecular assays analysis.

### 2.11 Histopathology

Lung tissues harvested from mice were fixed in 10% formalin, embedded in paraffin, sectioned at 7 μm, and stained with Hematoxylin and Eosin (H and E) staining, and Masson’s trichrome staining using commercial staining kits (Cat# D006 and Cat# D026, Nanjing Jiancheng Bioengineering Institute, Nanjing, China) according to the manufacturer’s instructions. These stainings are used to assess tissue architecture and lung fibrosis respectively. Blind scoring of fibrosis was conducted using Ashcroft’s scoring method [21] (0 to 8; 0 lowest, 8 highest) at 100× magnification.

### 2.12 Hydroxyproline Assay

Lung hydroxyproline content was quantified using the Hydroxyproline Assay Kit (Cat# A030-2, Nanjing Jiancheng Bioengineering Institute, Nanjing, China). In brief, lung tissues from mice were weighed, homogenized, and hydrolyzed in 12N HCl at 110°C overnight. After neutralization with citrate-acetate buffer (0.05 M), chloramine-T (0.05 M) was added and incubated for 20 min, followed by Ehrlich’s reagent (provided in the Hydroxyproline Assay Kit) for 15 min at 65°C. Absorbance was recorded at 550 nm wavelength using a Bio-Rad iMark Microplate Absorbance Reader (Model # 1681135, Bio-Rad Laboratories, Hercules, CA, USA). Data were expressed as μg hydroxyproline per mg lung tissue.

### 2.13 Myeloperoxidase (MPO) Activity

MPO activity in lung homogenates was quantified using the MPO Assay Kit (Cat# A044, Nanjing Jiancheng Bioengineering Institute, Nanjing, China). In brief, lung tissue was homogenized in 50 mM phosphate buffer (pH 6.0) with 0.5% HTAB and centrifuged at 12,000 rpm for 20 min. Supernatant was collected and MPO activity was measured using 3,3^′^-dimethoxybenzidine (0.17 mg/mL) and 0.0005% H_2_O_2_. Results were expressed as enzymatic units per gram of total protein.

### 2.14 Quantitative Reverse Transcription PCR (qRT-PCR)

Total RNA was extracted from lung tissues of mice using TRIzol Reagent (Cat# 15596026, Invitrogen, Thermo Fisher Scientific, Waltham, MA, USA). Reverse transcription was performed using PrimeScript RT Master Mix (Cat# RR036A, Takara Bio. Inc., Shiga, Japan) to synthesize cDNA. Quantitative PCR (qPCR) was carried out using TB Green Premix Ex Taq^TM^ II (Cat# RR820A, Takara Bio Inc., Shiga, Japan) on a CFX96 Touch Real-Time PCR Detection System (Bio-Rad Laboratories, Hercules, CA, USA). GAPDH was used for internal control. Primer sequences are as follows:

GAPDH (F: 5^′^-CCATCACTGCCACCCAGA-3^′^; R: 5^′^-ATGACCTTGCCCACAGCCTTG-3^′^);

CXCL1 (F: 5^′^-GATTCACCTCAAGAACATCCAGA-3^′^; R: 5^′^-GGACACCTTTTAGCATCTTTTGG-3^′^);

CXCL2 (F: 5^′^-AACATCCAGAGCTTGAGTGTGAC-3^′^; R: 5^′^-GCCTTGCCTTTGTTCAGTATCTT-3^′^);

TNF-α (F: 5^′^-CAGCCGATTTGCTATCTCATACC-3^′^; R: 5^′^-GTACTTGGGCAGATTGACCTCAG-3^′^).

### 2.15 Flow Cytometry

Lung tissues from mice were minced and digested with collagenase D (1 mg/mL) and DNase I (0.1 mg/mL) in Hank’s Balanced salt solution (HBSS; 1×, pH = 7.4) at 37°C for 30 min. Mixture was filtered (40 μm) to obtain single-cell suspension and Red blood cells (RBCs) were lysed with Pharm Lyse buffer (BD Biosciences, San Jose, CA, USA). Cells were blocked with 5% BSA on ice to reduce nonspecific binding and stained with PE-conjugated F4/80 antibody (Cat# 12-4801-82, 1:100; Thermo Fisher Scientific, Waltham, MA, USA) for 30 min at 4°C in dark. The gating strategy involved selecting singlets and viable cells based on forward and side scatter, followed by quantification of F4/80+ macrophages. A consistent gating approach was applied across all experimental groups. For apoptosis, A549 and H460 cells were stained with Annexin-V-FITC/PI Apoptosis Detection Kit (Cat# KGA107, KeyGEN Biotech, Nanjing, Jiangsu, China) after 48 h of gefitinib with or without G31P treatment. Analysis was performed using BD LSRII Flowcytometer (Model # 342974, BD Biosciences, San Jose, CA, USA) and using FlowJo software, version 11.0.2 (BD Biosciences).

### 2.16 Statistical Analysis

Data were presented as mean ± SEM. One-way ANOVA was used for comparing multiple groups, and Student’s *t-*test was applied for two-group comparisons. A *p*-value ≤ 0.05 was considered statistically significant. Analyses and graphs were generated using GraphPad Prism v 8.03 software (GraphPad Software, Boston, MA, USA).

## 3 Results

### 3.1 Gefitinib Combined with G31P Inhibits Proliferation and Migration of Lung Cancer Cells

**Figure 1:**
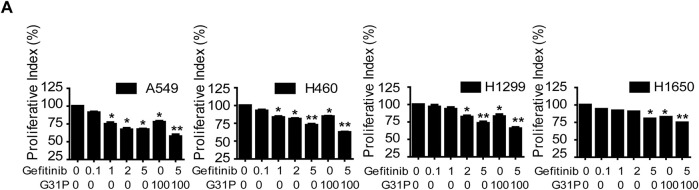

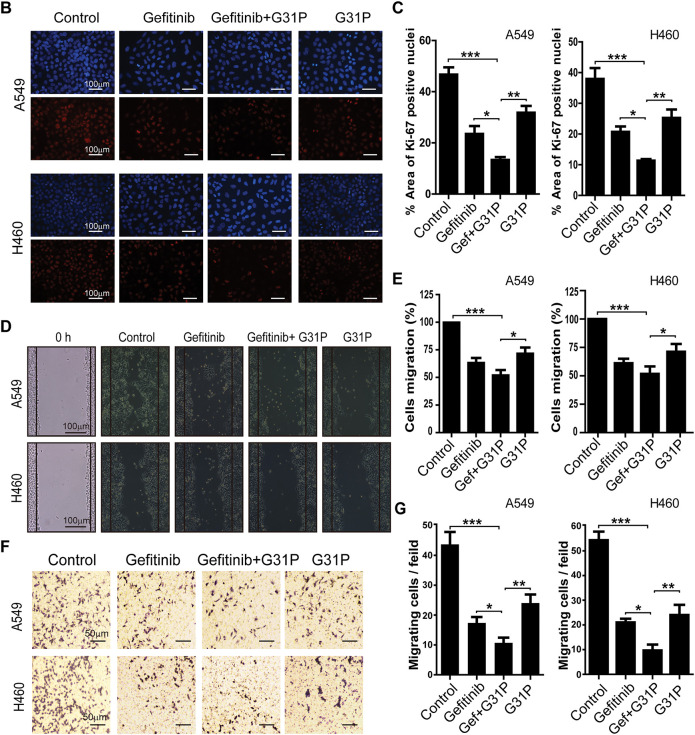
G31P and gefitinib combined treatment inhibits cell proliferation and migration in NSCLC. (**A**) Cells were treated with gefitinib at different concentrations (0.1, 1, 2, 5 μM) and/or 100 ng/mL of G31P for 48 h. Cell proliferation of A549, H460, H1299, and H1650 was measured by CCK8 assay at 450 nm. (**B**) Immunofluorescence images of Ki-67 expression (red color staining) in A549 and H460 cells treated with 5 μM of gefitinib and 100 ng/mL of G31P for 48 h, either alone or combined. DAPI (blue color staining) was used for nuclear staining. (**C**) Graph showing the percentage of area with positive Ki-67 staining (mean ± SEM) from three independent experiments. (**D**,**E**) Migration of A549 and H460 cells after 24 h was assessed in a wound healing assay after gefitinib (5 μM) +/− G31P (100 ng/mL) treatment. Bar graphs show the mean value of migration rate (%) from 3 independent experiments. (**F**,**G**) Cell transmigration was calculated by transwell assay after cells were exposed to gefitinib (5 μM) +/− G31P (100ng/mL). Graphs show the number of migrated cells over 24 h. All experiments were repeated three times. (*p < 0.05; **p < 0.01; ***p < 0.001)

Human NSCLC cell lines A549, H460, H1299, and H1650 were treated with varying concentrations of gefitinib, either alone or with G31P. Gefitinib monotherapy showed significant dose-dependent anti-proliferative effects across all four cell lines (*p* < 0.05 or *p* < 0.01 vs. untreated control; Fig. 1A). The addition of G31P (100 ng/mL) further increased the anti-proliferative effect in all tested cell lines (*p* < 0.01; Fig. 1A). Immunofluorescence analysis of Ki-67 expression in A549 and H460 cells corroborated these findings. The combined treatment with gefitinib and G31P significantly reduced nuclear Ki-67 expression compared to gefitinib alone (*p* < 0.05; Fig. 1B,C), confirming their synergistic anti-proliferative effects.

Cell migration was assessed by wound healing assays. A549 and H460 cells treated with gefitinib and G31P, showed a significantly increased wound closure compared to either drug alone or untreated controls (*p* < 0.05; Fig. 1D,E). Similarly, transwell migration assays demonstrated a significant inhibitory effect of transmigration of both cell lines with combined drug treatment compared to gefitinib (*p* < 0.05) or G31P (*p* < 0.01) monotherapy (Fig. 1F,G). These results collectively demonstrate that gefitinib combined with G31P significantly enhances the inhibition of lung cancer cell proliferation and migration *in vitro*.

### 3.2 Gefitinib and G31P Induce Apoptosis and Inhibit AKT and ERK Phosphorylation in Lung Cancer Cells

**Figure 2:**
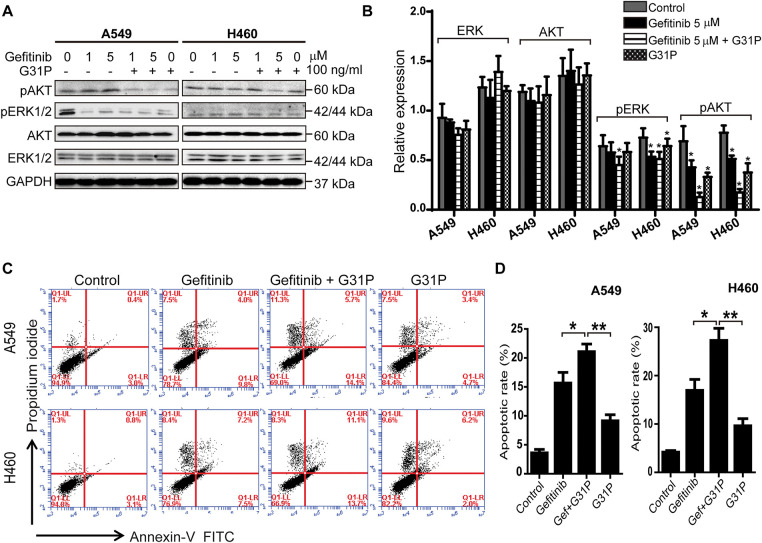
G31P and gefitinib treatment induce apoptosis and decrease pAKT and pERK1/2 in lung cancer cells. (**A**,**B**) A549 and H460 cells were treated with gefitinib (1, 5 μM) +/− G31P (100 ng/mL) for 24 h. Phosphorylation of AKT and ERK1/2 was detected by western blot, GAPDH was used as a loading control, normalized intensity is shown in bar graph. (**C**,**D**) A549 and H460 cells were treated with gefitinib (5 μM) +/− G31P (100 ng/mL) for 48 h. Cell apoptosis was measured by flow cytometry using Annexin-V-FITC/PI staining. The bar graph shows the apoptosis rate. All experiments were repeated three times. (*p < 0.05; **p < 0.01)

**Figure A1:**
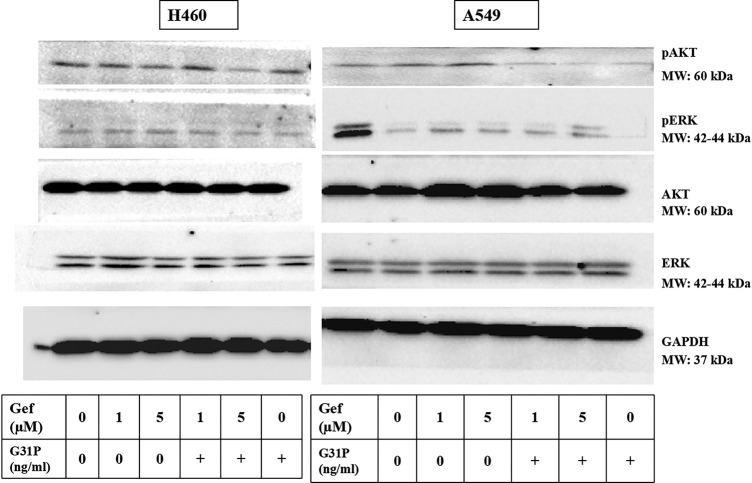
Western blot images of GAPDH, AKT, ERK, pERK, pAKT in H460 and A549 cells after treatment with gefitinib and G31P. “+” means with G31P treatment (100 ng/mL)

To determine the mechanism of anti-tumor effects, phosphorylation of key signaling pathways, AKT and ERK proteins, was assessed in A549 and H460 cells following treatment with gefitinib and/or G31P. Western blot analysis showed that the combination treatment significantly decreased AKT and ERK phosphorylation levels compared to either gefitinib or G31P alone (*p* < 0.05; Figs. 2A,B and A1). Notably, inhibition of pAKT was more pronounced than pERK inhibition, suggesting a prominent role of AKT pathway inhibition in mediating apoptosis.

Apoptosis was further evaluated using Annexin V/PI staining by flow cytometry. Gefitinib + G31P treatment significantly increased the proportion of apoptotic cells relative to gefitinib (*p* < 0.05) or G31P (*p* < 0.01) alone in both A549 and H460 cell lines (Fig. 2C,D). These data suggest that combined gefitinib and G31P treatment enhances its anti-proliferative effects and initiates apoptosis through suppression of AKT and ERK phosphorylation pathways.

### 3.3 Gefitinib Combined with G31P Inhibits Lung Cancer Growth in Mice Model

To evaluate the *in vivo* efficacy of gefitinib and its potential to induce pulmonary inflammation, a dose-tolerability assessment was performed in BALB/c nude mice. In a preliminary experiment, mice were treated with 200, 300, or 400 mg/kg of gefitinib orally for 5 days. The highest dose of gefitinib (400 mg/kg) induced adverse effects such as diarrhea, skin rash, and decreased physical activity. Based on these findings, 300 mg/kg was selected for further therapeutic experiments.

**Figure 3:**
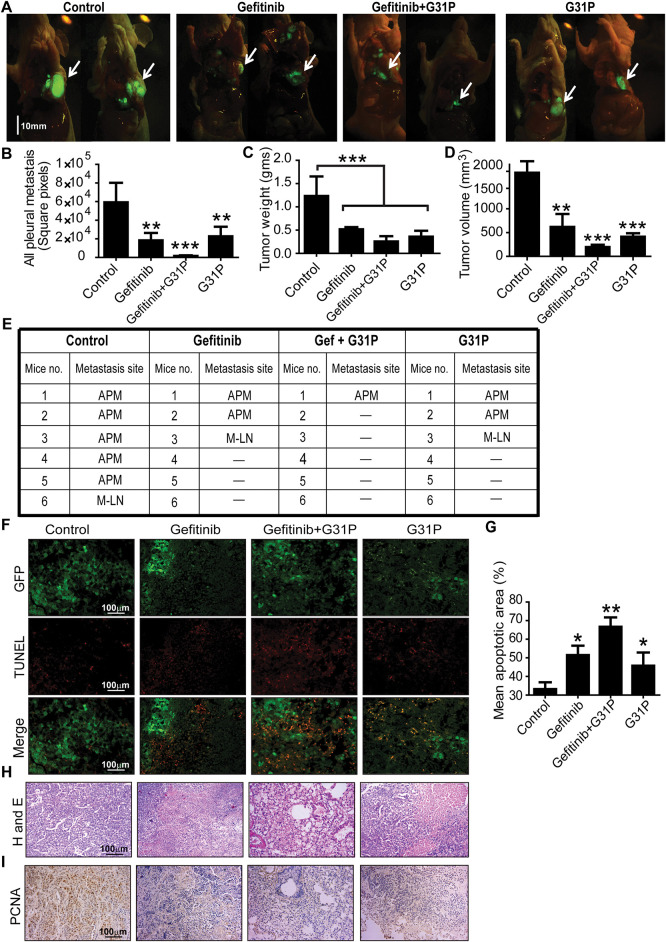
Combined G31P and gefitinib treatment inhibits H460 xenograft growth and metastasis in mice. (**A**) Fluorescent images show GFP-expressing H460 tumor cells from control, gefitinib, gefitinib + G31P, and G31P-treated mice. The arrow indicates the location of the orthotopic primary tumor, scale bar = 10 mm. **(B**) Metastasis was assessed by GFP square pixel area calculation outside the primary tumor. The graph represents the mean square pixel area of pleural cavity metastases. **(C**,**D**) Bar graphs show tumor weights and volumes. (n = 6/group). **(E**) Metastasis site in the pleural cavity of mice from all groups. “APM” = all pleural metastasis; “M-LN” = median lymph nodes; “-” = no metastasis observed. **(F**) Apoptosis was confirmed by TUNEL staining, wherein GFP fluorescence distinguished live and necrotic tumor cells. **(G**) Bar graph represents the mean apoptotic cell values calculated from five randomly chosen fields. (**H**) HE staining of tumor tissues from control and treated groups. (**I**) Immunohistochemistry images of PCNA expression in control and treated groups. (*p < 0.05; **p < 0.01; ***p < 0.001)

Orthotopic lung tumors were implanted in BALB/c nude mice using GFP-labeled H460 cells. After 28 days of treatment with gefitinib with or without G31P, tumor progression was monitored based on tumor weight, tumor volume, and GFP-detected metastasis (Fig. 3A). Pleural metastasis was significantly inhibited in all treatment groups, including gefitinib, G31P, and the combined gefitinib + G31P group, compared to control (*p* < 0.01; Fig. 3B,E). Notably, only 16.7% of mice in the gefitinib + G31P group developed pleural metastases, compared to 33.3% and 50% in the gefitinib and G31P monotherapy groups, respectively. Tumor weight was significantly decreased in all treated groups compared to the control group (Gefitinib, G31P vs. control, *p* < 0.01; Gefitinib + G31P vs. control, *p* < 0.001; Fig. 3C). Tumor volume was also significantly decreased in all treatment groups compared to control (*p* < 0.01; Fig. 3D).

TUNEL staining of tumor sections showed that gefitinib + G31P combined treatment induced significantly more apoptotic cells than monotherapies, while control group tumors showed the highest no. of GFP-positive viable cells (Fig. 3F,G). HE staining showed increased necrosis and fewer viable tumor cells in the combined treated group compared to others (Fig. 3H). PCNA immunohistochemistry showed that the gefitinib + G31P group significantly decreased proliferating cells compared to other groups (Fig. 3I). These results collectively indicate that G31P improves gefitinib-mediated anti-tumor effects *in vivo* by promoting apoptosis and suppressing tumor growth and metastasis.

### 3.4 G31P Decreases Gefitinib-Induced Pulmonary Inflammation in Tumor-Bearing Mice

**Figure 4:**
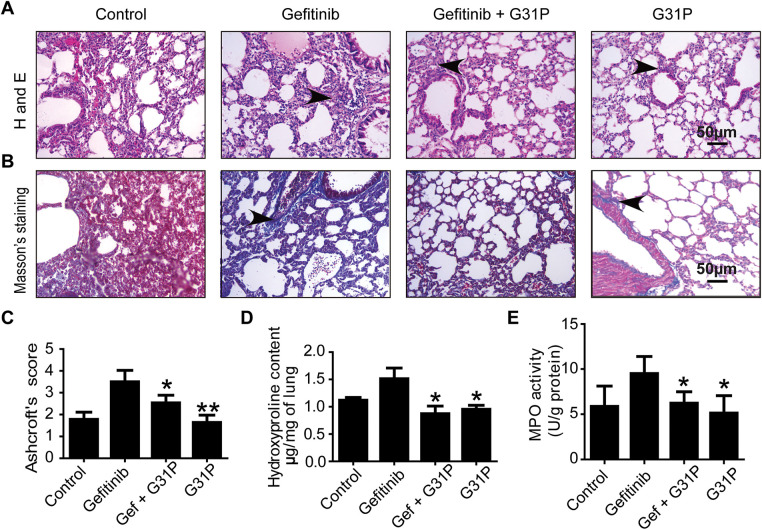

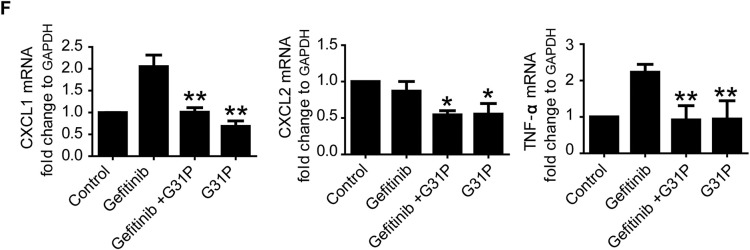
G31P decreases lung inflammation induced by gefitinib treatment in lung cancer mice. (**A**) HE staining of paraffin-embedded lung tissue slices showing inflammatory cell infiltration (arrowhead). (**B**) Masson’s trichrome staining of lung tissue sections in which blue staining represents collagen (arrowhead). (**C**) Graph of Ashcroft’s pulmonary fibrosis score from 0 (normal) to 8 (high-grade lung fibrosis). (**D**) Graph of hydroxyproline content of lung tissues (μg/mg tissue). (**E**) Graph of MPO content of lung tissues (U/g protein). (**F**) Bar graph of mRNA expression of inflammatory cytokines (CXCL1, CXCL2, and TNF-α) evaluated by qRT-PCR. GAPDH was used as an internal control. Experiments were repeated three times (*p < 0.05; **p < 0.01)

Left lung lobes from tumor-bearing mice were analyzed to assess pulmonary inflammation after treatment. Gefitinib-treated mice showed increased alveolar wall thickening and inflammatory cell infiltration, whereas these pathological features were improved in G31P-treated and combined-treatment groups (Fig. 4A). Masson’s trichrome staining showed higher collagen content in gefitinib-treated lungs, while the combination and G31P groups showed reduced fibrosis (Fig. 4B).

Ashcroft’s scoring showed a fibrosis severity of 5 in the gefitinib group, compared to 1 (control), 2 (G31P), and 3 (gefitinib + G31P), indicating partial reversal of gefitinib-induced fibrosis by G31P (*p* < 0.05, Fig. 4C). Lung hydroxyproline content followed this trend, confirming decreased collagen deposition in the combined-treatment group (*p* < 0.05, Fig. 4D). Similarly, MPO, a marker for neutrophil infiltration, was significantly inhibited in the G31P and combined-treatment groups compared to gefitinib alone (*p* < 0.05, Fig. 4E). Expression of pro-inflammatory cytokines CXCL1, CXCL2, and TNF-α was also significantly decreased in the G31P and combined treated groups compared to gefitinib monotherapy (Fig. 4F).

### 3.5 G31P Treatment Alleviates Inflammation in Bleomycin-Induced Lung Fibrosis

**Figure 5:**
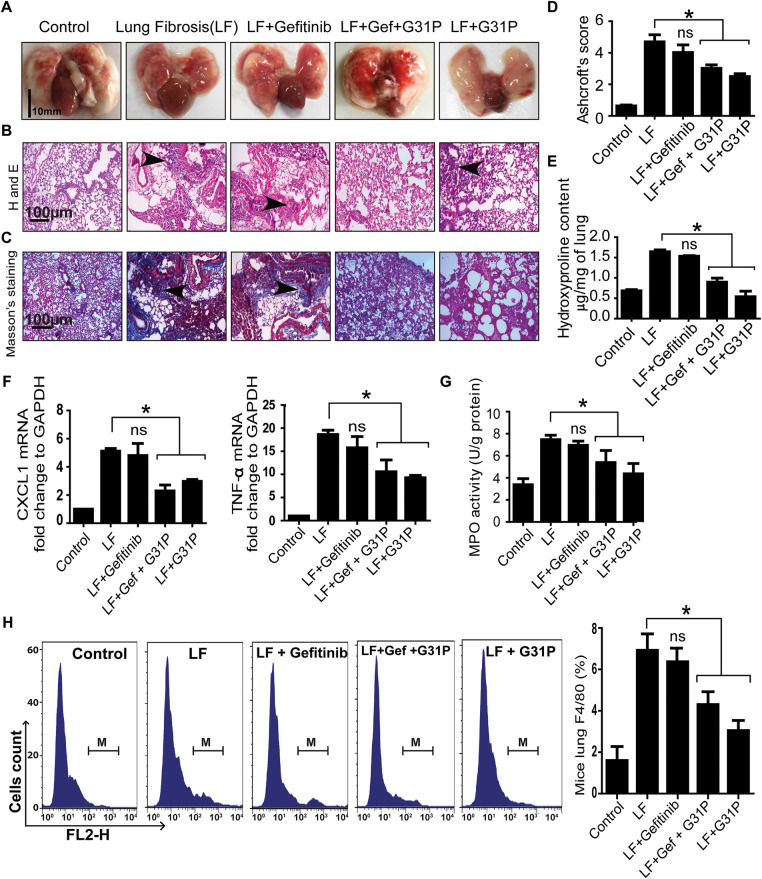
G31P decreases lung inflammation in bleomycin-induced lung fibrosis mice. (**A**) Images of lungs from control, lung fibrosis (LF), LF treated with gefitinib (300 mg/kg body weight), and/or G31P (0.5 mg/kg body weight). **(B**) HE staining of paraffin-embedded lung tissue sections showing inflammatory cell infiltration (arrowhead). **(C**) Masson’s trichrome staining of lung sections, where blue staining reveals tissue collagen, indicated by arrowhead. **(D**) Graph of Ashcroft’s pulmonary fibrosis scores, 0 (normal) to 8 (high-grade lung fibrosis). **(E**) Graph shows the hydroxyproline content (μg/mg tissue). **(F**) Graph of mRNA expression of inflammatory cytokines (CXCL1 and TNF-α) determined by qRT-PCR. GAPDH was used as internal control. **(G**) Graph of the MPO content of lung tissues (U/g protein). **(H**) Flow cytometry analysis of F4/80-positive inflammatory cells in lung tissue of mice. M represents the number of F4/80+ cells; graph shows the ratios of F4/80+ cells among total mononuclear cells. Experiments were repeated three times. (ns = non significant; *p < 0.05)

The bleomycin-induced lung fibrosis model was used to further confirm the anti-inflammatory potential of G31P. Lungs were harvested from mice and washed with PBS (Fig. 5A). Histopathology showed that G31P treatment inhibited inflammatory cell infiltration and preserved alveolar structure. In contrast, gefitinib worsened lung injury, with alveolar damage and thickened parenchyma (Fig. 5B). Masson’s trichrome staining showed higher collagen deposition in gefitinib-treated lungs, while G31P and combined-treatment groups showed decreased collagen content compared to the untreated lung fibrosis group (Fig. 5C). Aschoft’s scores were 6 for the fibrosis control group, 5 for the gefitinib group, 3 for the gefitinib + G31P group, and 2.5 for the G31P group, respectively. These scores confirm the protective effects of G31P (Fig. 5D). Hydroxyproline content quantification also supported these observations, showing that G31P alone or in combination with gefitinib significantly inhibited collagen content compared to fibrosis controls and gefitinib alone (Fig. 5E).

Similarly, lung inflammatory cytokine levels of CXCL1, TNF-α, and MPO were significantly decreased by G31P alone or in combination with gefitinib compared to the fibrosis control (*p* < 0.05), while no significant difference was observed between the gefitinib and fibrosis control groups (Fig. 5F,G). Flow cytometry results showed a significantly decreased proportion of F4/80-positive macrophages in G31P and combined drug-treated groups vs. the fibrosis control (*p* < 0.05), while there were no significant differences between the gefitinib and fibrosis control groups (Fig. 5H). These findings demonstrate that G31P has a potent anti-inflammatory effect on lung injury by inhibiting fibrosis and immune cells infiltration, even in the presence of gefitinib.

## 4 Discussion

Gefitinib is widely used to treat NSCLC because of its ability to suppress tumor cell proliferation and promote apoptosis through inhibition of downstream pathways, such as PI3K/AKT and MAPK/ERK [22,23]. However, its clinical use is often limited by adverse effects such as ILD and pneumonitis, particularly in Asian populations [24]. These toxicities have also been observed in preclinical models, in which gefitinib treatment causes cytokine upregulation and inflammatory cell infiltration [25,26]. In severe cases of EGFR-TKI induced pneumonitis, the therapeutic approach is either drug withdrawal or treatment with corticosteroids to reduce side effects. However, corticosteroids carry risks including systemic immunosuppression, metabolic complications, and potential interference with anticancer immunity, while dose reduction may compromise antitumor efficacy in advanced disease [27]. Therefore, drugs that ameliorate EGFR-TKI side effects without compromising their therapeutic efficacy are desired for combined application. Consistent with these findings our results showed that gefitinib-treated mice showed increased levels of cytokine, collagen deposition, and hydroxyproline content in lung tissue, but combined treatment with G31P significantly ameliorated these pathological changes demonstrating its potential to decrease gefitinib-induced pulmonary damage.

G31P is a synthetic form of CXCL8 with modifications in lysine to arginine (K11R) and glycine to proline (G31P) substitutions, which confer potent CXCR1/2 antagonism, thereby blocking ELR+ CXC chemokines signaling pathways involved in tumor growth, neutrophil infiltration, and resistance to therapy [20,28]. Previous studies have reported its efficacy in mice models of prostate, liver, lung and breast cancers, where it suppressed tumor growth and attenuated inflammatory cytokine release [29]. Our findings are consistent with these results, as G31P inhibited CXCL1, CXCL2 and TNF-α in tumor and bleomycin-induced lung tissues, alongside decreased MPO activity and F4/80+ macrophage infiltration. In addition, G31P-mediated CXCR1/2 inhibition disrupts the pathological feedback loop in the tumor microenvironment, where ELR+ CXC chemokines (CXCL1/8) activate CXCL8, leading to EGFR transactivation and sustained inflammatory signaling. This feedback is particularly relevant in EGFR-TKI-resistant tumors, where CXCL8/CXCR2 pathways are upregulated [30,31]. Our results align with this mechanistic understanding, as G31P potentiated gefitinib efficacy while blocking inflammatory cytokine expression without affecting its treatment efficacy.

G31P has shown promising therapeutic potential in combination with various treatments in different disease models. For example, G31P combined with cisplatin has been reported to protect against cisplatin-induced nephrotoxicity by decreasing renal inflammation [28]. In a breast cancer mice model, G31P combined with GDC-0941 improved anti-tumor efficacy through modulation of tumor-promoting cytokines and the tumor microenvironment [32]. In a diabetic mice model, G31P treatment provided nephroprotective effects by reducing inflammation and fibrosis associated with diabetic nephropathy [33]. G31P also inhibits pulmonary inflammation in LPS-challenged mice or in swine burn dust-instilled mice by inhibiting CXCL1/8 [34]. These diverse protective effects emphasize G31P’s versatility as an adjuvant drug capable of improving therapeutic efficacy while mitigating treatment-related toxicities in different disease settings.

Mechanistically, our results indicate that the enhanced anti-tumor effects observed with gefitinib + G31P treatment are primarily due to inhibition of PI3K/AKT and MAPK/ERK pathways, which are central to EGFR-driven tumor growth [35]. The significant suppression of AKT phosphorylation suggests that G31P may potentiate the apoptotic signaling initiated by gefitinib, consistent with the proposed crosstalk between CXCR1/2 and EGFR signaling pathways [36]. G31P inhibits AKT and ERK phosphorylation by directly blocking CXCR1/2 signaling in tumor cells and indirectly decreases CXCL1/8 driven paracrine activation, consistent with evidence that CXCR1/2 blockade inhibits PI3K/AKT signaling in multiple cancers. It also suppresses neutrophilic inflammation and cytokine production. Together, these effects suggest that G31P modulates both cancer cell-intrinsic pathways and immune-mediated pathways and thereby increasing cancer cell sensitivity to therapy and promoting apoptosis.

To further confirm the effects of G31P in lung injury, we used another mice model of lung fibrosis induced by intratracheal instillation of bleomycin, which cause alveolar and interstitial pneumonitis progressing to pulmonary fibrosis. Damage to alveolar epithelial cells results in the release of inflammatory cytokines and growth factors that stimulate myofibroblasts and pathological extracellular matrix secretion, leading to fibrosis. Previous studies have reported that fibrogenic cytokines including CXCL1, CXCL2, TNF-α, and CXCL8 are involved in bleomycin-induced pneumonitis [37]. Consistent with this, we observed that mice receiving bleomycin intratracheally exhibited high levels of inflammatory cytokine including CXCL1, TNF-α along with increased collagen deposition in the lungs. These inflammatory cytokines were not decreased after gefitinib treatment, which causes activation of macrophages and neutrophils in the lung fibrosis model. In contrast, mice treated with G31P showed markedly lower cytokine levels, MPO activity and F4/80-positive macrophages in lung tissue compared to untreated lung fibrosis induced mice, indicating reduced immune reactions.

In addition, both orthotopic lung cancer and bleomycin lung fibrosis models strengthen the translational relevance of our findings. The bleomycin model mimics the pathophysiology of chemotherapy-induced pneumonitis and fibrosis [38], and our data demonstrate that G31P inhibited fibrosis markers and prevented inflammatory cell infiltration and mediators expression. These findings support the inclusion of G31P in therapeutic regimens to reduce off-target organ toxicities and indicate that it deserves further investigation.

Despite the promising findings, this study has several limitations that should be acknowledged. Most of the experimental work was performed mainly in cell lines and mice model, which cannot fully reproduce the complexity of human NSCLC or the heterogeneity of patient responses. In addition, while our data suggest that CXCR1/2 inhibition enhances the antitumor efficacy of gefitinib and reduces pulmonary inflammation, the precise molecular mechanisms underlying the crosstalk between CXCR1/2 blockade and EGFR signaling remain to be fully elucidated. Moreover, clinical factors such as patient genetic background, pre-existing pulmonary diseases, or comorbidities were not addressed in our mice models and may influence therapeutic outcomes. Addressing these aspects in future mechanistic and translational studies, including well-designed clinical trials, will be essential to confirm the therapeutic potential of G31P as an adjuvant to EGFR-TKIs therapy.

## 5 Conclusion

This study provides evidence that the CXCR1/2 antagonist G31P enhances the anti-tumor efficacy of gefitinib and protects against chemotherapy-induced lung inflammation and fibrosis. G31P has a dual action: it suppresses tumor-promoting chemokine signaling and mitigates pulmonary toxicity, making it a promising adjuvant for EGFR-TKI-based therapies. These findings support further preclinical and clinical investigations of G31P as a multifunctional therapeutic agent to improve the efficacy and safety of NSCLC treatment.

## Data Availability

All data or analysis during this study are included in this manuscript.

## References

[1] Mengistu BA, Tsegaw T, Demessie Y, Getnet K, Bitew AB, Kinde MZ, et al. Comprehensive review of drug resistance in mammalian cancer stem cells: implications for cancer therapy. Cancer Cell Int. 2024;24(1):406. doi:10.1186/s12935-024-03558-0; 39695669 PMC11657890

[2] Elmore LW, Greer SF, Daniels EC, Saxe CC, Melner MH, Krawiec GM, et al. Blueprint for cancer research: critical gaps and opportunities. CA Cancer J Clin. 2021;71(1):7–29. doi:10.3322/caac.21652; 33326126

[3] Bayat Mokhtari R, Homayouni TS, Baluch N, Morgatskaya E, Kumar S, Das B, et al. Combination therapy in combating cancer. Oncotarget. 2017;8(23):38022–43. doi:10.18632/oncotarget.16723; 28410237 PMC5514969

[4] O’Leary C, Gasper H, Sahin KB, Tang M, Kulasinghe A, Adams MN, et al. Epidermal growth factor receptor (EGFR)-mutated non-small-cell lung cancer (NSCLC). Pharmaceuticals. 2020;13(10):273. doi:10.3390/ph13100273; 32992872 PMC7600164

[5] Ohmori T, Yamaoka T, Ando K, Kusumoto S, Kishino Y, Manabe R, et al. Molecular and clinical features of EGFR-TKI-associated lung injury. Int J Mol Sci. 2021;22(2):792. doi:10.3390/ijms22020792; 33466795 PMC7829873

[6] Skribek M, Rounis K, Tsakonas G, Ekman S. Complications following novel therapies for non-small cell lung cancer. J Inter Med. 2022;291(6):732–54. doi:10.1111/joim.13445; 35032058

[7] Miyake K, Tani K, Kakiuchi S, Suzuka C, Toyoda Y, Kishi J, et al. Epidermal growth factor receptor-tyrosine kinase inhibitor (gefitinib) augments pneumonitis, but attenuates lung fibrosis in response to radiation injury in rats. J Med Invest. 2012;59(1–2):174–85. doi:10.2152/jmi.59.174; 22450006

[8] Shah RR. Tyrosine kinase inhibitor-induced interstitial lung disease: clinical features, diagnostic challenges, and therapeutic dilemmas. Drug Saf. 2016;39(11):1073–91. doi:10.1007/s40264-016-0450-9; 27534751

[9] Kirkil G, Mogulkoc N, Jovanovic D. Risk factors and management of lung cancer in idiopathic pulmonary fibrosis: a comprehensive review. Sarcoidosis Vasc Diffuse Lung Dis. 2025;42(1):15604; 40100103 10.36141/svdld.v42i1.15604 PMC12013682

[10] Komolafe K, Pacurari M. CXC chemokines in the pathogenesis of pulmonary disease and pharmacological relevance. Inter J Inflam. 2022;2022(1):4558159. doi:10.1155/2022/4558159; 36164329 PMC9509283

[11] Boon K, Vanalken N, Szpakowska M, Chevigné A, Schols D, Van Loy T. High-affinity ELR+ chemokine ligands show G protein bias over β-arrestin recruitment and receptor internalization in CXCR1 signaling. J Biol Chem. 2025;301(1):108044. doi:10.1016/j.jbc.2024.108044; 39615686 PMC11732455

[12] Do HT, Lee CH, Cho J. Chemokines and their receptors: multifaceted roles in cancer progression and potential value as cancer prognostic markers. Cancers. 2020;12(2):287. doi:10.3390/cancers12020287; 31991604 PMC7072521

[13] Zhou C, Gao Y, Ding P, Wu T, Ji G. The role of CXCL family members in different diseases. Cell Death Discov. 2023;9(1):212. doi:10.1038/s41420-023-01524-9; 37393391 PMC10314943

[14] Mishra A, Suman KH, Nair N, Majeed J, Tripathi V. An updated review on the role of the CXCL8-CXCR1/2 axis in the progression and metastasis of breast cancer. Mol Biol Rep. 2021;48(9):6551–61. doi:10.1007/s11033-021-06648-8; 34426905

[15] Asokan S, Bandapalli OR. CXCL8 signaling in the tumor microenvironment. Adv Exp Med Biol. 2021;1302(3):25–39. doi:10.1007/978-3-030-62658-7_3; 34286439

[16] Matsushima K, Yang D, Oppenheim JJ. Interleukin-8: an evolving chemokine. Cytokine. 2022;153(4897):155828. doi:10.1016/j.cyto.2022.155828; 35247648

[17] Liu X, Wang C, Mao H, Wei J. Crosstalk between cancer-associated fibroblasts and inflammation in tumor microenvironment: a novel perspective in cancer therapy. Oncol Rep. 2025;54(2):93; 40511592 10.3892/or.2025.8926 PMC12183503

[18] Sitaru S, Budke A, Bertini R, Sperandio M. Therapeutic inhibition of CXCR1/2: where do we stand? Intern Emerg Med. 2023;18(6):1647–64. doi:10.1007/s11739-023-03309-5; 37249756 PMC10227827

[19] Cheng HT, Yu HY, Gordon JR, Li F, Cheng JW. Effects of K11R and G31P mutations on the structure and biological activities of CXCL8: solution structure of human CXCL8_(3-72)_K11R/G31P. Molecules. 2017;22(7):1229. doi:10.3390/molecules22071229; 28754019 PMC6152285

[20] Khan MN, Wang B, Wei J, Zhang Y, Li Q, Luan X, et al. CXCR1/2 antagonism with CXCL8/interleukin-8 analogue CXCL8_(3-72)_K11R/G31P restricts lung cancer growth by inhibiting tumor cell proliferation and suppressing angiogenesis. Oncotarget. 2015;6(25):21315–27. doi:10.18632/oncotarget.4066; 26087179 PMC4673267

[21] Hübner RH, Gitter W, Eddine El Mokhtari N, Mathiak M, Both M, Bolte H, et al. Standardized quantification of pulmonary fibrosis in histological samples. Biotechniques. 2008;44(4):507–17. doi:10.2144/000112729; 18476815

[22] Han S, Tian Z, Tian H, Han H, Zhao J, Jiao Y, et al. HDGF promotes gefitinib resistance by activating the PI3K/AKT and MEK/ERK signaling pathways in non-small cell lung cancer. Cell Death Discov. 2023;9(1):181. doi:10.1038/s41420-023-01476-0; 37301856 PMC10257651

[23] Zhao ZQ, Yu ZY, Li J, Ouyang XN. Gefitinib induces lung cancer cell autophagy and apoptosis via blockade of the PI3K/AKT/mTOR pathway. Oncol Lett. 2016;12(1):63–8. doi:10.3892/ol.2016.4606; 27347100 PMC4906680

[24] Shi J, Liu X, Gao M, Yu J, Chai T, Jiang Y, et al. Adverse event profiles of EGFR-TKI: network meta-analysis and disproportionality analysis of the FAERS database. Front Pharmacol. 2025;16:1519849. doi:10.3389/fphar.2025.1519849; 40135231 PMC11933087

[25] Terasaki Y, Suzuki T, Tonaki K, Terasaki M, Kuwahara N, Ohsiro J, et al. Molecular hydrogen attenuates gefitinib-induced exacerbation of naphthalene-evoked acute lung injury through a reduction in oxidative stress and inflammation. Lab Invest. 2019;99(6):793–806. doi:10.1038/s41374-019-0187-z; 30710119

[26] Noguchi T, Sekiguchi Y, Kudoh Y, Naganuma R, Kagi T, Nishidate A, et al. Gefitinib initiates sterile inflammation by promoting IL-1β and HMGB1 release via two distinct mechanisms. Cell Death Dis. 2021;12(1):49. doi:10.1038/s41419-020-03335-7; 33414419 PMC7791030

[27] Teuwen LA, Van den Mooter T, Dirix L. Management of pulmonary toxicity associated with targeted anticancer therapies. Expert Opin Drug Metab Toxicol. 2015;11(11):1695–707. doi:10.1517/17425255.2015.1080687; 26293379

[28] Li L, Khan MN, Li Q, Chen X, Wei J, Wang B, et al. G31P, CXCR1/2 inhibitor, with cisplatin inhibits the growth of mice hepatocellular carcinoma and mitigates high-dose cisplatin-induced nephrotoxicity. Oncol Rep. 2015;33(2):751–7. doi:10.3892/or.2014.3659; 25504010

[29] Jeong Y, Yoon SY, Jung SP, Nam SJ, Lee JE, Kim S. Inhibition of interleukin-8/CXC chemokine receptor 2 signaling axis prevents tumor growth and metastasis in triple-negative breast cancer cells. Pharmacology. 2025;110(3):178–90. doi:10.1159/000545659; 40188812 PMC12105825

[30] Ha H, Debnath B, Neamati N. Role of the CXCL8-CXCR1/2 axis in cancer and inflammatory diseases. Theranostics. 2017;7(6):1543–88. doi:10.7150/thno.15625; 28529637 PMC5436513

[31] Huang X, Hao J, Tan YQ, Zhu T, Pandey V, Lobie PE. CXC chemokine signaling in progression of epithelial ovarian cancer: theranostic perspectives. Int J Mol Sci. 2022;23(5):2642. doi:10.3390/ijms23052642; 35269786 PMC8910147

[32] Li X, Zhang Y, Walana W, Zhao F, Li F, Luo F. GDC-0941 and CXCL8_(3-72)_K11R/G31P combination therapy confers enhanced efficacy against breast cancer. Future Oncol. 2020;16(14):911–21. doi:10.2217/fon-2020-0035; 32285685

[33] Cui S, Zhu Y, Du J, Khan MN, Wang B, Wei J, et al. CXCL8 antagonist improves diabetic nephropathy in male mice with diabetes and attenuates high glucose-induced mesangial injury. Endocrinology. 2017;158(6):1671–84. doi:10.1210/en.2016-1781; 28387853

[34] Wang X, Li Y, Li L, Jiao Z, Liu X, Cheng G, et al. Porcine CXCR1/2 antagonist CXCL8_(3-72)_G31P inhibits lung inflammation in LPS-challenged mice. Sci Rep. 2020;10(1):1210. doi:10.1038/s41598-020-57737-w; 31988368 PMC6985246

[35] Stefani C, Miricescu D, Stanescu-Spinu I-I, Nica RI, Greabu M, Totan AR, et al. Growth factors, PI3K/AKT/mTOR and MAPK signaling pathways in colorectal cancer pathogenesis: where are we now? Int J Mol Sci. 2021;22(19):10260. doi:10.3390/ijms221910260; 34638601 PMC8508474

[36] Tang KH, Li S, Khodadadi-Jamayran A, Jen J, Han H, Guidry K, et al. Combined inhibition of SHP2 and CXCR1/2 promotes antitumor T-cell response in NSCLC. Cancer Discov. 2022;12(1):47–61. doi:10.1101/2021.03.21.436338. 34353854 PMC8758507

[37] Chan Y, Raju Allam VS, Paudel KR, Singh SK, Gulati M, Dhanasekaran M, et al. Nutraceuticals: unlocking newer paradigms in the mitigation of inflammatory lung diseases. Crit Rev Food Sci Nutr. 2023;63(19):3302–32. doi:10.1080/10408398.2021.1986467; 34613853

[38] Xu C, Shang Z, Najafi M. Lung pneumonitis and fibrosis in cancer therapy: a review on cellular and molecular mechanisms. Curr Drug Targets. 2022;23(16):1505–25. doi:10.2174/1389450123666220907144131; 36082868

